# Where were those rabbits? A new paradigm to determine cerebral lateralisation of visuospatial memory function in children

**DOI:** 10.1016/j.neuropsychologia.2011.07.031

**Published:** 2011-10

**Authors:** Margriet A. Groen, Andrew J.O. Whitehouse, Nicholas A. Badcock, Dorothy V.M. Bishop

**Affiliations:** a Department of Experimental Psychology, University of Oxford, South Parks Road, Oxford OX1 3UD, United Kingdom; b Behavioural Science Institute, Radboud University Nijmegen, P. O. Box 9104, 6500 HE Nijmegen, The Netherlands; c Telethon Institute for Child Health Research, Centre for Child Health Research, University of Western Australia, 100 Roberts Road, Subiaco 6008, Western Australia, Australia

**Keywords:** Cerebral lateralisation, Visuospatial, Right hemisphere, Functional transcranial Doppler ultrasonography, Children

## Abstract

In the majority of people, functional differences are observed between the two cerebral hemispheres: language production is typically subserved by the left hemisphere and visuospatial skills by the right hemisphere. The development of this division of labour is not well understood and lateralisation of visuospatial function has received little attention in children. In this study we devised a child-friendly version of a paradigm to assess lateralisation of visuospatial memory using functional transcranial Doppler ultrasound (fTCD). In a group of 24 adults we found this child-friendly version gave similar results to the original version of the task. In addition, fourteen children aged 6–8 years successfully completed the child-friendly fTCD task, showing a negative lateralisation index, indicating right hemispheric specialisation at the group level. Additionally, we assessed effects of task accuracy and reaction time on the lateralisation index. No effects were found, at the group level or at the level of single trials, in either the adult or the child group. We conclude that this new task reliably assesses lateralisation of visuospatial memory function in children as young as 6 years of age, using fTCD. As such, it holds promise for investigating development of lateralisation of visuospatial function in typically and atypically developing children.

## Highlights

► A child-friendly fTCD task to measure cerebral lateralisation of visuospatial memory. ► Strong positive correlations were found between the child and adult versions. ► No associations between lateralisation and task performance were found. ► It was a valid measure of cerebral lateralisation for visuospatial memory in children.

## 1 Introduction

In the majority of people, functional differences are observed between the two cerebral hemispheres: language production is typically subserved predominantly by the left hemisphere and visuospatial skills by the right hemisphere. Originally derived from patient studies, this finding has been replicated using a variety of imaging techniques, including the Wada test and functional magnetic resonance imaging (Springer & Deutsch, 1993).

Little is known about why, how and when humans arrive at this pattern of cerebral lateralisation. A better understanding of the development of typical patterns of lateralisation of language and other cognitive functions is of both theoretical and practical interest. Insight into the development of laterality patterns by studying typically developing children may add to our understanding of the origins of this division of labour, as well as shedding light on mechanisms underpinning atypical lateralisation in clinical groups. Deviations from the typical pattern of left and right hemispheric specialisation for language and visuospatial function, respectively, have been reported in individuals with early brain injuries due to epilepsy (Helmstaedter, Kurthen, Linke, & Elger, 1997; Pataraia et al., 2004) or stroke (Stiles, Reilly, Paul, & Moses, 2005). A higher than normal rate of atypical hemispheric specialization for language function has also been reported in people with a neurodevelopmental disorder such as dyslexia (Illingworth & Bishop, 2009; Shaywitz et al., 2007; Temple, 2002), specific language impairment (Bernal & Altman, 2003; Chiron et al., 1999; Shafer, Schwartz, Morr, Kessler, & Kurtzberg, 2000; Tzourio, Heim, Zilbovicius, Gerard, & Mazoyer, 1994; Whitehouse & Bishop, 2008), developmental stuttering (Wood, Stump, McKeehan, Sheldon, & Proctor, 1980), and Down syndrome (Bowler, Cufflin, & Kiernan, 1985; Elliott & Weeks, 1993; Hartley, 1981; Pipe, 1983).

At present, our knowledge of the development of the typical pattern of functional lateralisation comes from studies employing behavioural measures such as hand preference, visual half-field techniques or dichotic listening (for a review, see Kinsbourne, 2009). These techniques show weak to moderate correlations with cerebral lateralisation as determined by the Wada test (Bishop, 1990; Pelletier, Sauerwein, Lepore, Saint-Amour, & Lassonde, 2007) and are less accurate in detecting right-hemisphere and bilateral language representation (Channon, Schugens, Daum, & Poldey, 1990; Kurthen, 1992). In the past 15 years, functional magnetic resonance imaging (fMRI) has become more popular for use in paediatric samples and has been used to address the issue of lateralisation of language (Gaillard et al., 2000, 2003; Hertz-Pannier et al., 1997; Holland et al., 2001, 2007; Szaflarski, Schmithorst, et al., 2006; Szaflarski, Holland, Schmithorst, & Byars, 2006; Wood et al., 2004) and visuospatial (Everts et al., 2009; Thomason et al., 2009) function in children. However, several drawbacks of using fMRI in this context remain. First, due to drop-out rates as high as 40–50% at young ages (Byars et al., 2002; Holland et al., 2001) and the considerable expense of scanning (Pelletier et al., 2007), sample sizes are often small, with a few notable exceptions (Holland et al., 2007; Szaflarski, Holland, et al., 2006). Furthermore, due to movement restrictions language tasks are typically covert and often involve considerable meta-linguistic skills.

In recent years functional transcranial Doppler ultrasonography (fTCD) has been shown to be a reliable, non-invasive and relatively inexpensive methodology for determining cerebral lateralisation (Deppe, Ringelstein, & Knecht, 2004). Because fTCD is quick to set up, is relatively insensitive to movement, and can be carried out in a quiet and comfortable environment, the technique has great potential for assessing cerebral lateralisation in children. Indeed, children as young as 2 years of age have been found to be able to comply with the procedure (Lohmann, Drager, Müller-Ehrenberg, Deppe, & Knecht, 2005).

Several groups have developed and evaluated tasks that probe lateralisation for language suitable for children (Bishop, Watt, & Papadatou-Pastou, 2009; Haag et al., 2010; Lohmann et al., 2005; Stroobant, Van Boxstael, & Vingerhoets, 2011). These involve either the description of pictures or animations (expressive tasks), or listening to stories (receptive task). Both types of tasks result in lateralisation to the left hemisphere in the majority of cases, but this is more pronounced for the expressive tasks (Stroobant et al., 2011). Test reliabilities for these tasks are reported as good to excellent (Bishop et al., 2009; Haag et al., 2010; Lohmann et al., 2005; Stroobant et al., 2011).

Lateralisation of visuo-spatial function in children has received less attention and has not, to the best of our knowledge, been investigated in children using fTCD, and no suitable task for children has been reported to date. In adults, tasks probing visuo-spatial attention (Flöel, Buyx, Breitenstein, Lohmann, & Knecht, 2005; Flöel et al., 2001; Hartje, Ringelstein, Kistinger, Fabianek, & Willmes, 1994; Lust, Geuze, Groothuis, & Bouma, 2011; Vingerhoets & Stroobant, 1999) or visuospatial memory (Whitehouse, Badcock, Groen, & Bishop, 2009; Whitehouse & Bishop, 2009) have resulted in greater right than left hemisphere activation as assessed with fTCD.

The aims of the current study were twofold. Firstly, to evaluate the sensitivity of a fTCD paradigm for assessing cerebral lateralisation for visuospatial memory in children. To this end, we adapted a visuospatial memory task that has resulted in clear right-lateralised activation in adults (Whitehouse & Bishop, 2008) and which has shown good reproducibility (Whitehouse et al., 2009). We compared this new child-friendly version with the original version in adults and also report on its use with a group of school-aged children. Secondly, we investigated whether behavioural performance affected lateralisation scores. One possibility is that children are less lateralised than adults as a result of less proficient performance on the task at hand. Additionally, given that reaction times show considerable development during childhood and adolescence, the time-course of cognitive processes and associated lateralised activation may also differ. We assessed these issues by investigating associations between task accuracy and reaction time and the size and direction of the laterality index both at the group level and at the level of single trials.

## 2 Materials and methods

### 2.1 Participants

Adult participants (*n* = 24, 17 women, aged between 18–29 years, *M* = 22.25, SD = 3.32) were staff and students of Oxford University, all of whom were part of a larger sample recruited for a previous study (Whitehouse & Bishop, 2009). In the previous study participants were administered a spatial memory task along with a word generation task that determines laterality for language. Selected members of this sample were contacted again for the current study, with a particular emphasis on recruiting those individuals with atypical (i.e., left-hemisphere) lateralisation for the spatial memory task, in order to provide an evaluation of the full range of cerebral activation. Six further potential participants were seen but did not produce enough useable trials in the Circles paradigm (3 cases) or the Rabbits paradigm (3 cases) due to technical error (2 cases) or poor signal due to non-optimal probe placement or failure to locate the temporal window (4 cases).

Child participants were 14 typically developing children (6 girls) aged 6–8 years (*M* = 7.01 years, SD = 0.44 years), recruited from primary schools around Oxfordshire. Six further children were seen but were excluded from the analyses because not enough useable trials were recorded due to technical failure (1 case), failure to comply with the task (1 case), or difficulty locating the temporal window or non-optimal probe placement (4 cases).

Participants in both groups were healthy, without any history of neurological disorder and with normal or corrected to normal vision. Handedness was assessed with the Edinburgh Handedness Inventory^1^ (Oldfield, 1971), with scores of 40 or above denoting right-handedness, 40 or below denoting left-handedness, and scores in between denoting ambidexterity. The sample included 15 right-handed (11 women), 8 left-handed (6 women) and 1 ambidextrous (men) adults and 9 right-handed (3 girls), 1 left-handed (girl) and 4 ambidextrous (2 girls) children.

All adult participants gave written informed consent, whereas parental consent and child assent were obtained for the 6–8-year-old children. The project was approved by the Central University Research Ethics Committee of the University of Oxford and is in accordance with the WMA Declaration of Helsinki for experiments involving humans.

### 2.2 Apparatus

Blood flow velocity through the left and right middle cerebral arteries (MCA) was measured with a Doppler ultrasonography device (DWL Multidop T2: manufacturer, DWL Elektronische Systeme, Singen, Germany). Participants were fitted with a flexible head-set, which held in place a 2-MHz transducer probe over each temporal skull window. The spatial memory paradigms were controlled by Presentation Software (Neurobehavioral Systems) on a Dell laptop computer, which sent markers to the fTCD to denote the start of each epoch. Responses during the Rabbits paradigm were given via a Microtouch touch screen (3M Touch Systems, Bracknell, UK).

### 2.3 Stimuli

The *Circles paradigm* was identical to the spatial memory paradigm outlined in Whitehouse and Bishop (2008). In short, each trial started with a cueing tone and a ‘clear mind’ message was displayed on the screen for an initial 5-s interval. Then white (*n* = 17) and red (*n* = 9) circles appeared on the screen, overlaid on a black background. The circles were distributed approximately evenly across the screen, but were not aligned in rows or columns. Participants were instructed to memorize the location of the red circles, which were randomly located around the screen. The circles remained on the screen for 5 s, and were then replaced by a blank screen for 10 s. Following another cueing tone, the circle array appeared again. In half of the 20 trials, the location of the red (and white) circles was the same, while in the other half of the trials, the location of one white and one red circle was swapped. Participants, who sat with their hands on the table in front of them, were asked to decide whether the location of the red circles was the same or different as the initial screen, by raising the index finger on their left or right hand, respectively. This was followed by a 25 s rest period. ‘Same’ and ‘different’ trials were in the same random order for all participants. Participants were awarded one point for each trial that they correctly identified as the ‘same’ or ‘different’.

In the *Rabbits paradigm* each trial started with a cueing tone and a ‘clear mind’ message was displayed on the screen for an initial 5-s interval. Then 20 black circles (‘the holes’) appeared on a green background for the Rabbits paradigm used with adults. Similarly to the circles in the Circles paradigm, the ‘holes’ were distributed approximately evenly across the screen, but were not aligned in rows or columns. Six out of 20 holes had a white rabbit in them. Participants were instructed to memorize which holes had a rabbit in them. The holes and rabbits remained on the screen for 5 s, and were then replaced by a blank screen for 10 s. Following another cueing tone, the holes re-appeared and the participant was asked to indicate which holes had had a rabbit in them in the previous screen by touching those holes on a touch screen. The trial ended after the participant had touched 6 holes. This was followed by a 25 s rest period. The locations of the holes were the same on all trials, while the locations of the rabbits varied across trials. The same random locations were used for each participant. Participants completed 2 blocks of 12 (adults) or 10 (children) trials responding with their left hand in one block and their right hand in the other block. Block order and response hand were counterbalanced across participants. A practice trial preceded the first block. After piloting the adult version of the Rabbits paradigm with children, it was adapted so that: (1) The initial display of holes with rabbits in them was shown for 4, not 5 s; and (2) the following blank screen only remained for 6, not 10 s. Finally, the numbers of holes and rabbits was varied to create five levels of difficulty. The easiest level showed seven holes, two of which had a rabbit in them, the most difficult level was identical to the one used with the adult participants (20 holes, 6 rabbits). Intermediate levels had ten, thirteen, or seventeen holes, three, four, or five of which had a rabbit in them, respectively. Children completed a practice run prior to the experimental blocks in which 2 trials were presented at each difficulty level. For the experimental blocks the child was presented with the highest difficulty level at which he or she located all rabbits correctly on at least 1 of the 2 trials during the practice run.

### 2.4 Procedure

Adult participants were tested in a quiet laboratory and completed both paradigms in one session. Child participants were tested in a quiet laboratory, a separate room in their school or at home and completed only the Rabbits paradigm.

### 2.5 Data analysis

Data from each fTCD paradigm were analysed using dopOSCCI (Badcock, Holt, Holden, & Bishop, in preparation), which is a Matlab script (Mathworks Inc., Sherborn, MA, USA) written by one of the authors (NB). The following steps were carried out: (1) the blood ﬂow envelope from each probe was downsampled to 25 Hz, (2) heart beat activity was removed by determining local peaks in the signal from the left probe and using the heart cycle integration described by Deppe, Knecht, Henningsen, & Ringelstein, (1997), (3) in order to control for global differences in recorded velocity, unrelated to the task, between the left and the right probe, blood ﬂow values were normalised to a mean of 100% on a trial-by-trial basis. Time-locked epochs were then averaged, after rejecting epochs with unusually high or low levels of activity (±30% of the average blood flow velocity in adults, ±40% in children), as a result of non-optimal probe positioning or excessive movement. For both groups and both paradigms, trials during which the participant was not “on task” (e.g., not paying attention, talking during the baseline) were also excluded from the analysis. Using these combined criteria, about 90% of trials was retained (91 and 88% for adults on the Circles and the Rabbits task, respectively; 92% for children). All adult participants had at least 12 (out of 20) accepted epochs on the Circles and 15 (out of 24) accepted epochs on the Rabbits paradigm. Pilot work suggested that this results in a valid indication of cerebral lateralisation for visuospatial memory with these tasks. On average, more trials were included for the Rabbits (*M* = 21.08, SD = 1.44) than for the Circles paradigm (*M* = 18.17, SD = 1.13; *t*(23) = −10.60, *p* < 001, *r* = .91) for adults. Children had at least 14 (out of 20) accepted epochs on the Rabbits paradigm (*M* = 18.43, SD = 1.95). The mean difference curve for left and right channels was corrected to give a mean value of zero over a baseline period of 10 s prior to the presentation of the stimulus.

A laterality index (LI) was calculated as the mean blood flow velocity difference in a 2-s window centred on the peak difference value during the period of interest. The period of interest was based on previous work with the Circles paradigm (Whitehouse & Bishop, 2009) in combination with visual inspection and occurred during the recognition/remembering phase for both paradigms (Adults: Circles and Rabbits 22–37 s after the start of the trial; Children: Rabbits 20–35 s). The LI latency refers to the time in seconds of the peak left minus right difference, relative to trial onset. In addition to the LI based on the average of all epochs for a participant, the LI and its latency were also extracted for single trials. This enabled us to evaluate effects of task performance and reaction time on the LI on a single-trial basis. For the Rabbits paradigm, trials used to calculate the LI were balanced in terms of response hand (i.e., the same number of trials responded to with each hand were included). A positive LI indicated greater left than right hemisphere activation, with a negative index signifying the reverse. To assess reliability, split-half reliabilities were calculated by computing the LI values for odd and even epochs, and correlating these.

## 3 Results

### 3.1 Adults

#### 3.1.1 Assessing lateralisation of visuospatial memory using a child-friendly task

**Fig. 1:**
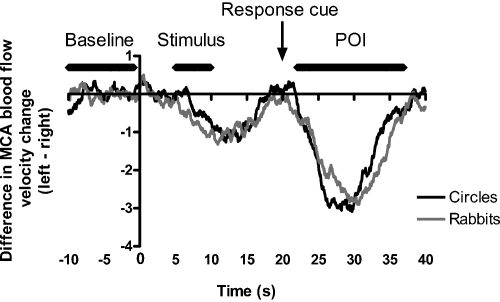
Mean difference waveforms for adult data on the Circles (black) and the Rabbits (grey) paradigm. The time points where the stimulus appeared (Stimulus), part of the stimulus reappeared and the response cue (Response cue) was given, and the period of interest (POI) for calculation of the laterality index is also indicated.

Mean difference curves (cerebral blood flow velocity change in the left minus the right channel) for the Circles and the Rabbits paradigm are plotted in Fig. 1. The curves looked similar, as was confirmed by the strong and statistically significant positive intra-class correlation coefficient (ICC)^2^, *r* = .91, *p* < .001. Table 1 summarizes the LIs and their latencies, as well as the *t*-value for testing difference from zero. For both paradigms the LI was negative and significantly different from zero, indicating lateralisation to the right hemisphere at the group level. Paired *t*-tests did not show significant differences between the Circles and the Rabbits paradigm with regard to the LIs (*t*(23) = 0.44, *p* = .663) or the latency at which the LIs occurred (*t*(23) = 1.52, *p* = .144).

**Fig. 2:**
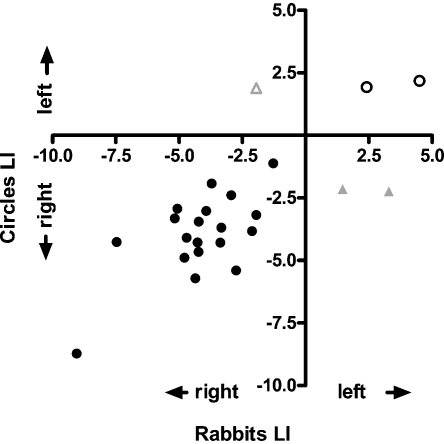
Scatterplot of adult laterality indices (LIs) for the Circles and the Rabbits paradigm. Black dots represent individuals who showed right- (filled dots) or left- (open dots) lateralised activation on both paradigms. Grey triangles represent individuals who showed right-lateralised activation on the Circles paradigm, but left-lateralised activation on the Rabbits paradigm (filled triangles) or vice versa (open triangle).

Fig. 2 shows a scatterplot of each participant's LI for the two paradigms. Visual inspection suggests a high-level of congruence between the LIs for the two paradigms, as was confirmed by a significant positive Pearson's correlation coefficient, *r*(24) = .75, *p* < 001. As well as computing a LI, it is possible to categorise a participant as being left- or right-lateralised or show bilateral activation, using the standard error of the LI across epochs to determine if the 95% confidence interval of that individual's LI overlaps with zero. When considered in this manner, 87.5% of participants were right-lateralised and 12.5% left-lateralised on the Circles paradigm. On the Rabbits paradigm, 83.3% were right-lateralised and 16.7% left-lateralised. Overlap between the categorisations based on the two paradigms was high: twenty-one (87.5%) individuals were in the same category for both paradigms (19 right-lateralised, 2 left-lateralised). The remaining individuals were categorised as right-lateralised on the Circles paradigm, but as left-lateralised (2) on the Rabbits paradigm or vice versa (1).

Table 2 summarizes accuracy (Circles and Rabbits paradigm) and reaction time (Rabbits paradigm only) data. For the Circles and the Rabbits paradigm, the average percent correct across trials (Avg) is reported. For the Rabbits paradigm only, the percentage of trials on which participants located all rabbits correctly (Trials all correct, Tac) is also reported. Adult participants were highly accurate on both paradigms. Although adults registered 92% correct responses across all trials, they remembered all six locations on each trial correctly on only ∼68% of trials on average, ranging from ∼29% to 95% of trials. This leaves some scope to examine the influence of task performance on the LI.

#### 3.1.2 The influence of task performance on the LI

First we looked at the time course of both the behavioural and physiological responses. We found a statistical trend for participants who more often located all rabbits on a trial correctly to take less time to respond, as indicated by a negative correlation between Tac and Duration (*r*(24) = −.38, *p* = .064). When looking at the single-trial level, we observed a similar relationship. Because the single-trial data were skewed, non-parametric correlations were inspected. On trials where a participant responded more accurately, (i.e., a higher percentage of rabbits was located correctly), the first response was faster (*τ* = −.22, *p* < .001) and the total duration of manual responses was shorter (*τ* = −.32, *p* < .001). To assess a possible association between the time point at which the LI occurred and the time point of the manual response on the single-trial level, we looked for correlations between these variables for trials on which all rabbits were located correctly (Tac = 100%, *n* = 373). No significant correlations were observed.

**Fig. 3:**
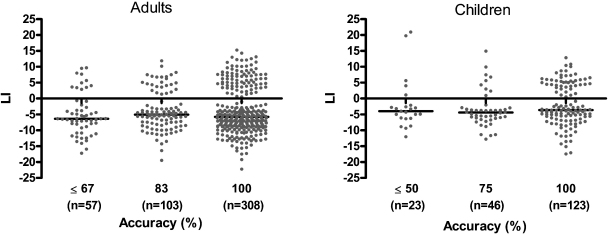
Single-trial data for the LI for individuals categorised as showing right-lateralised activation on the Rabbits task, across trials at which: adults located ≤67, 83 or 100% of rabbits correctly (left panel), or children located ≤50, 75 or 100% of rabbits correctly (right panel). Each dot represents the LI on a single trial. Horizontal lines indicate the mean LI at a particular accuracy level. The number of trials included at a given accuracy level are in parentheses.

Second, we assessed associations between task accuracy and the direction and size of the LI. No differences in accuracy were found between people who were right- or left-lateralised on the respective tasks (Circles: *t*(20) = 0.02, *p* = .984; Rabbits: Avg *t*(22) = 1.21, *p* = .238, Tac *t*(22) = 1.62, *p* = .119). On the single-trial level for people who showed right-lateralised activation, no significant differences in the size of the LI were found at different accuracy levels (*H*(2) = 2.05, *p* = .359; Fig. 3).

### 3.2 Children

#### 3.2.1 Assessing lateralisation of visuospatial memory

The mean LI was −1.78 (SD = 2.02, range −5.78 to 1.64), which is significantly different from zero on a *t*-test (*t*(13) = −3.30, *p* = .006), indicating lateralisation to the right hemisphere at the group level. The mean latency of the LI was 25.65 (SD = 3.87). The odd-even split-half reliability was medium, *r* = .53, *p* = .05. When considered categorically, 11 cases (78.6%) were right-lateralised and 3 cases (21.4%) were left-lateralised.

Twelve children completed the Rabbits task at level 4 (13 holes, 4 rabbits), whereas the remaining 2 children completed level 5 (17 holes, 5 rabbits). Accuracy levels and reaction times across the group as a whole are summarized in Table 2. Child participants were highly accurate on the Rabbits paradigm, registering 85% correct responses across all trials on average. However, they remembered all locations on a trial correctly on only ∼59% of trials on average, ranging from ∼5% to 100% of trials. This leaves some scope to examine the influence of task performance on the LI.

#### 3.2.2 The influence of task performance on the LI

To assess the influence of task performance on the LI, we first looked at the timecourse of both the behavioural and physiological responses. We found that children who more often located all rabbits on a trial correctly, took less time to respond as indicated by a significant negative correlation between Tac and Duration (*r*(14) = −.56, *p* = .037). When looking at the single-trial level, we found a similar relationship. Because the single-trial data were skewed, non-parametric correlations were inspected. On trials where a higher percentage of rabbits was located correctly, the first response was faster (*τ* = −.15, *p* = .001) and the total duration of manual responses was shorter (*τ* = −.34, *p* < .001). To assess a possible association between the timepoint at which the LI occurred and the timepoint of the manual response on the single-trial level, we looked for correlations between these variables for trials on which all rabbits were located correctly (Tac = 100%, *n* = 142). No significant correlations were observed.

Secondly, we assessed associations between task accuracy and the direction and size of the LI. At the group level no differences in accuracy were found between children who were categorized as right- or left-lateralised (Avg *t*(12) = −0.65, *p* = .527, Tac *t*(12) = −0.94, *p* = .365). On the single-trial level, for children who showed right-lateralised activation, no significant differences in the size of the LI were found at different accuracy levels (*H*(2) = 2.83, *p* = .243; Fig. 3).

## 4 Discussion

Whitehouse and Bishop (2008) presented a task that has shown good reproducibility in determining hemispheric specialisation for visuospatial memory function using fTCD in adults (Whitehouse et al., 2009). In the current study, we compared this task with a child-friendly version and obtained highly similar results in adults. The Circles and the Rabbits task resulted in highly similar activation curves (Fig. 1), and LIs obtained by the two tasks for the same participant were strongly associated (Fig. 2). Most adults (87.5%) ended up in the same category (right-lateralised, left-lateralised) for both paradigms. However, two participants showed right-lateralised activation on the Circles task but left-lateralised activation on the Rabbits task, and a third exhibited the reverse pattern. A similar finding was observed in the study by Whitehouse et al. (2009), in which 2 of 30 participants (6.67%) had different patterns of lateralisation on the Circles task at two different time points. These discrepancies may reflect participants’ lack of attention on one or both of the tasks, or the use of different cognitive strategies by these participants between tasks. It is also possible that, just as with handedness, some people are able to perform the task competently with either hemisphere. Importantly, however, these participants were in the minority, with the Rabbits task successfully identifying the correct pattern of visuospatial lateralisation with close to 90% reliability.

Children successfully completed the fTCD procedure and most found the visuospatial memory task enjoyable. In 75% of children, clean data on cerebral lateralisation of visuospatial memory function could be obtained. Similar to adults, we found a negative LI in the children, indicating right hemispheric specialization at the group level. Split-half reliability was satisfactory and comparable to that obtained in adults.

No effects of task performance on the direction or the size of the LI were found, neither at the overall or single trial level, nor within the adult or child groups. It appears that as long as the participant was ‘on task’ (trials during which this was not the case were excluded from the analysis), lateralised activation was observed in both adults and children. This finding is in accordance with recent studies in adults (Lust, Geuze, Groothuis, & Bouma, 2011; Lust, Geuze, Groothuis, van der Zwan, et al., 2011) in which no relationship between task performance and cerebral lateralisation on a spatial task was found under single-task conditions. Given that both children and adults achieved a high level of accuracy on the Rabbits task, it could also be that the limited variation in task performance hampered the detection of such effects. Similarly, the timepoint of the manual response as measured by the first response or the duration of all responses, did not affect the latency of the LI on the Rabbits paradigm. Possibly the blood flow velocity response is too sluggish to be sensitive to small changes in the timecourse of cognitive processes.

In conclusion, this new task reliably assesses lateralisation of visuospatial memory function in children as young as 6 years of age, using fTCD. Because of the quick, non-invasive and relatively low-cost nature of fTCD, it holds promise to investigate development of lateralisation of visuospatial function in typically and atypically developing children.

## Figures and Tables

**Table 1:** Mean laterality indices and their latencies, with *t*-value for testing difference from zero for adult participants in the two paradigms. Split-half reliability is also included.

	Circles	Rabbits
*N*	24	24
LI		
*M*	−3.06	−2.87
SD	2.48	3.16
Range	−8.72 to 2.18	−9.05 to 4.50
*t*	−6.06**	−4.45**
Split-half reliability	.56*	.63**
Latency		
*M*	23.96	23.84
SD	0.48	0.57

* *p* < .05.

** *p* ≤ .001.

**Table 2:** Mean accuracy and reaction time for adults and children. Avg = average across trials; Tac = trials all correct.

	Adults		Children
	Circles	Rabbits	Rabbits
Accuracy			
Avg (%)			
*M*	88.41	92.33	85.23
SD	6.62	4.81	9.72
Range	70.00–95.00	79.86–99.31	67.00–100.00
Tac (%)			
*M*		68.41	58.57
SD		16.48	24.61
Range		29.17–95.83	5.00–100.00
Reaction time			
First			
*M*		2109.58	2230.34
SD		934.43	348.39
Range		1254.50–5641.70	1702.60–3065.00
Duration			
*M*		6394.69	6487.35
SD		1407.33	1069.49
Range		4520.85–10844.00	5216.65–8433.35

## Footnotes

1 Because the item “striking match” was considered inappropriate for children, we replaced it for them with an item asking which hand they used to deal playing cards.

2 The ICC is conceptually similar to the Pearson correlation coefficient, but is sensitive to the position, as well as the shape, of the two curves.
